# Two-Year Clinical Outcomes of Critical Limb-Threatening Ischemia Versus Claudication After Femoropopliteal Endovascular Therapy: An Analysis from K-VIS ELLA Registry

**DOI:** 10.3390/jcm14248919

**Published:** 2025-12-17

**Authors:** Soohyung Park, Markz R. M. P. Sinurat, Seung-Woon Rha, Byoung Geol Choi, Se Yeon Choi, Cheol Ung Choi, Young-Guk Ko, Donghoon Choi, Jae-Hwan Lee, Chang-Hwan Yoon, In-Ho Chae, Cheol Woong Yu, Seung Whan Lee, Seung Hyuk Choi, Pil-Ki Min, Chang Gyu Park, on behalf of the K-VIS (Korean Vascular Intervention Society) Investigators

**Affiliations:** 1 Cardiovascular Center, Korea University Guro Hospital, Seoul 08308, Republic of Korea; theblue_rose@korea.ac.kr (S.P.); markzroland91@gmail.com (M.R.M.P.S.);; 2 Cardiovascular Research Institute, Korea University, Seoul 02841, Republic of Korea; 3 Department of Biomedical Laboratory Science, Honam University, Gwangju 62399, Republic of Korea; 4 Division of Cardiology, Department of Internal Medicine, Severance Cardiovascular Hospital, Yonsei University College of Medicine, Seoul 03722, Republic of Korea; 5 Division of Cardiology, Department of Internal Medicine, Chungnam National University Hospital, Daejeon 35015, Republic of Korea; 6 Division of Cardiology, Seoul National University Bundang Hospital, Seongnam 13620, Republic of Korea; 7 Division of Cardiology, Department of Internal Medicine, Korea University Anam Hospital, Seoul 02841, Republic of Korea; 8 Division of Cardiology, Department of Internal Medicine, Asan Medical Center, University of Ulsan College of Medicine, Seoul 05505, Republic of Korea; 9 Division of Cardiology, Department of Medicine, Samsung Medical Center, Sungkyunkwan University School of Medicine, Seoul 06351, Republic of Korea; 10 Division of Cardiology, Department of Internal Medicine, Gangnam Severance Hospital, Yonsei University College of Medicine, Seoul 03722, Republic of Korea

**Keywords:** peripheral artery disease, critical limb-threatening ischemia, claudication, endovascular, femoropopliteal artery disease

## Abstract

**Background/Objectives**: Endovascular therapy (EVT) is the treatment of choice for femoropopliteal artery (FPA) disease manifesting as critical limb-threatening ischemia (CLTI) or intermittent claudication (IC). This study aimed to compare the clinical outcomes of patients with CLTI with those of patients with IC after EVT in a real-world setting. **Methods**: In total, 1924 patients with FPA disease (CLTI: n = 812, IC: n = 1112) from the K-VIS ELLA (Korean Vascular Intervention Society Endovascular Therapy in Lower Limb Artery Diseases) registry who underwent EVT between 2006 and 2021 were analyzed. The primary endpoint was defined as target limb amputation or clinically driven (CD) target extremity revascularization (TER) 2 years after the procedure. **Results**: The incidence of the primary endpoint after inverse probability of treatment weighting (IPTW) was significantly higher in the CLTI group (hazard ratio [HR], 1.314; 95% confidence interval [CI], 1.105–1.561; *p* = 0.002). The incidences of loss of clinical patency, major adverse limb events (MALEs), and all-cause mortality were also higher in the CLTI group (hazard ratio [HR], 1.312; 95% confidence interval [CI], 1.157–1.488; *p* < 0.001). However, the risk of repeat percutaneous transluminal angioplasty (PTA) was similar between the groups (HR, 1.014; 95% CI, 0.833–1.234; *p* = 0.920). The use of drug-coated balloons (DCBs) was associated with favorable primary outcomes in both groups, particularly in patients with IC (HR: 0.429, 95% CI: 0.25–0.734; *p* = 0.002). **Conclusions**: Patients with CLTI undergoing EVT for FPA disease experienced worse clinical outcomes than those with IC, although the repeat PTA rates were similar. The use of DCBs showed promising results in both groups.

## 1. Introduction

Peripheral artery disease (PAD), a common manifestation of advanced systemic atherosclerosis, is the third leading cause of atherosclerotic morbidity after coronary heart disease and stroke [1,2]; it affects over 200 million adults globally. Atherosclerotic changes in the femoropopliteal artery (FPA) often result in the formation of long occlusive calcified lesions [3]. FPA disease may remain asymptomatic or present with symptoms ranging from intermittent claudication (IC) to severe ischemic conditions such as tissue loss or gangrene [2]. Critical limb-threatening ischemia (CLTI), a severe form of PAD, is characterized by resting pain, non-healing wounds, and tissue damage [4]. Endovascular therapy (EVT) is widely accepted as the preferred revascularization strategy for femoropopliteal artery disease, particularly in high-risk surgical risk [5]. Rapid advancements have been made in EVT for the management of FPA disease in recent years, and a significant improvement in post-treatment clinical outcomes has been observed since the introduction of newer treatment devices such as drug-coated balloons (DCBs) and drug-eluting stents (DESs) for the management of FPA disease [6,7,8,9,10].

The comorbidities observed in patients with CLTI are typically worse than those observed in patients with claudication. Moreover, patients with CLTI are at a higher risk of adverse events such as major amputations and periprocedural complications. Notably, a higher risk of cardiovascular events, including increased mortality [4,11], has been observed in patients with CLTI. Thus, it is imperative to develop an optimal treatment strategy to improve outcomes in this high-risk population is imperative [4,12,13]. However, few studies have directly compared the outcomes of EVT in patients with CLTI and those with IC. Therefore, this study aimed to compare the clinical outcomes of EVT in patients with native FPA disease according to the presence of CLTI or IC, using data from a real-world multicenter cohort with a 2-year follow-up period.

## 2. Materials and Methods

### 2.1. Data Sources

The Korean Vascular Intervention Society Endovascular Therapy in Lower Limb Artery Diseases (K-VIS ELLA) is a multicenter registry that includes data from patients diagnosed with lower extremity PAD who underwent EVT at 19 cardiovascular centers in South Korea between 2006 and 2021 (ClinicalTrials.gov: NCT02748226).

### 2.2. Population

**Figure 1:**
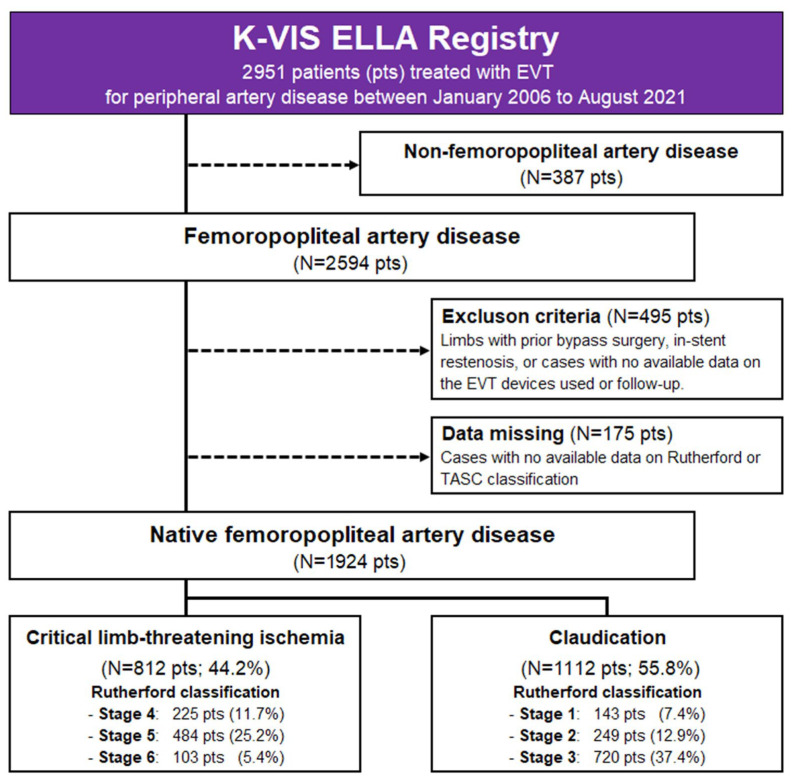
Study flowchart. EVT, endovascular therapy; K-VIS ELLA, Korean Vascular Intervention Society Endovascular Therapy in Lower Limb Artery Diseases.

The study design and inclusion/exclusion criteria are detailed in a previous publication [3]. Figure 1 presents a flowchart of the study. Among the 2951 patients included in the registry, 2564 who had undergone EVT for FPA disease were eligible for inclusion in the present study. Patients who underwent bypass surgery, those with in-stent restenosis, and those with no data available regarding the EVT devices used, follow-up duration, Rutherford class, or TASC classification were excluded from the present study. Thus, the final sample comprised 1924 patients. The Institutional Review Board of each participating center approved the study protocol. This study was conducted in accordance with the tenets of the Declaration of Helsinki. Informed consent was obtained from all patients, except for those whose data were retrospectively collected. All the patients underwent a comprehensive clinical assessment at baseline. The clinical and imaging data of the patients, along with their demographic characteristics and comorbidities, were collected prospectively and analyzed retrospectively. Follow-up examinations were scheduled 6, 12, and 24 months after the initial procedure. The median follow-up duration was 1.9 ± 0.4 years. All patients were followed up for up to 2 years after EVT.

### 2.3. Procedures

Experienced interventional cardiologists performed all EVT procedures using the device of their choice. Intraluminal wiring was performed using 0.018- or 0.035-inch guidewires. A subintimal approach with re-entry into the distal true lumen or a retrograde approach was used in cases where wire passage could not be achieved. Pre-dilatation was performed using a plain balloon unless an atherectomy was initially performed. Pretreatment with atherectomy devices was favored in selected cases of calcified or long-segment lesions after intraluminal wire passage. The dilatation pressure of the DCB was determined by the surgeon and maintained above the nominal pressure. This pressure was maintained for at least 180 s after successful vessel preparation. Provisional stenting with bare metal stents (BMS) was performed if flow-limiting dissection or residual stenosis of ≥30% was angiographically confirmed after DCB, and these cases were included in the DCB group. Post-dilatation with a non-compliant balloon was performed after stenting with the BMS and DES to achieve a residual stenosis of <30%. Periprocedural complications such as vascular rupture, distal embolization, or access-site bleeding were managed at the operator’s discretion, typically with prolonged balloon inflation, additional stent implantation (including bailout stenting), thrombus aspiration or distal protection, and adjunctive pharmacologic therapy. Unless contraindicated, dual antiplatelet therapy with aspirin and P2Y12 inhibitors such as clopidogrel was initiated and continued for at least 6 months after the procedure. Cilostazol, a third antiplatelet agent, was administered at the physician’s discretion.

### 2.4. Definitions and Study Endpoints

The primary outcome was the target limb amputation or clinically driven (CD) target extremity revascularization (TER). CD-TER was defined as any subsequent intervention on the target extremity performed for stenosis > 50% of the angiographic diameter at a site within 5 mm proximal or distal to the original treatment segment, accompanied by symptom worsening and a decrease in ankle-brachial index (ABI) > 0.15 compared to the immediate postprocedural ABI. Loss of clinical patency, major adverse limb events (MALEs), and all-cause mortality were the secondary outcomes. Clinical patency was defined as the absence of worsening of symptoms by ≥1 Rutherford category change accompanied by a decrease of >0.15 in the ABI or a ≥50% absence of restenosis on imaging studies such as duplex ultrasound, computed tomographic angiography, or intra-arterial angiography. Above-ankle amputation or repeat revascularization of the index limb was defined as MALE. Revascularization of the target vessel with <30% residual stenosis and the absence of flow-limiting dissection was defined as technical success. Major amputation was defined as amputation of the index limb occurring above the ankle level, whereas minor amputation was defined as any amputation below the ankle, including toe or forefoot amputation. All patients were followed up for up to 2 years from the date of the procedure until the occurrence of an outcome event. The principal investigators at the participating centers identified and adjudicated all the clinical events.

### 2.5. Statistical Analysis

Continuous variables are presented as mean ± standard deviation, whereas discrete variables are presented as counts and percentages. The unpaired *t*-test or Mann–Whitney rank test was used to assess the differences between continuous variables. The χ^2^ test or Fisher’s exact test was used to assess the differences between discrete variables. Inverse probability weighting (IPTW) analysis was conducted using a logistic regression model to adjust for confounding factors. Table 1 and Table 2 present the covariates used to calculate the propensity for CLTI. Cox proportional regression analysis adjusted for IPTW was performed to determine the impact of various types of EVT on the primary and secondary endpoints over a 2-year follow-up period. All statistical analyses were performed using SPSS software (version 24.0; SPSS-PC Inc., Chicago, IL, USA). Statistical significance was defined as a two-sided *p*-value of <0.05.

## 3. Results

### 3.1. Baseline Characteristics

**Figure 2:**
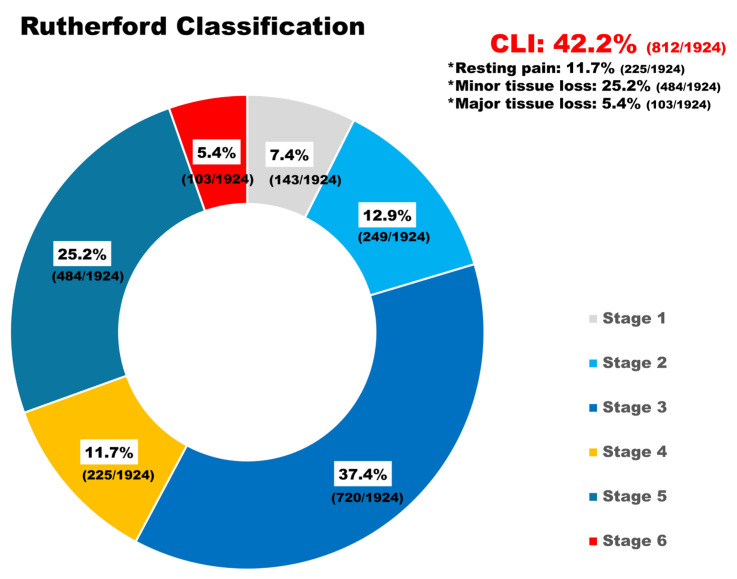
**Distribution of Rutherford classifications in the study population**. The donut chart illustrates the proportion of Rutherford classifications among 1924 patients who underwent femoropopliteal endovascular therapy. Critical limb-threatening ischemia (CLTI; stages 4–6) occurred in 44.2% of the study population.

Of the 1924 patients included in the final analysis, 812 (42.2%) were classified as having CLTI, and 1112 (57.8%) as having IC. Table 1 presents the baseline demographics, comorbidities, risk factors, prior surgical or intervention procedures, and laboratory findings of the patients with CLTI and IC before and after IPTW. The median age of the patients was 69.7 years (CLTI, 70.3; IC, 69.0), and 81.6% were male (CLTI, 81.7%; IC, 81.6%). Regarding severity, 37.4% of the IC group were categorized as Rutherford class 3, whereas 25.2% of the CLTI group were categorized as Rutherford class 5 (Figure 2). The sex distribution, age, BMI, and comorbidities, such as hypertension, diabetes, dyslipidemia, presence of coronary artery disease, and history of myocardial infarction (MI), were adequately balanced after IPTW.

Table 2 shows the angiographic and procedural characteristics, and the list of devices is shown in Supplementary Table S1. Among patients treated with DCB, the IN.PACT balloon (paclitaxel 3.5 µg/mm^2^ with urea excipient, Medtronic, Minneapolis, MN, USA) was most frequently used, accounting for more than 70% of all cases, followed by Lutonix (paclitaxel 2.0 µg/mm^2^ with polysorbate/sorbitol excipient, Bard Peripheral Vascular, Tempe, AZ, USA) and Ranger (paclitaxel 2.0 µg/mm^2^ with acetyl tributyl citrate excipient, Boston Scientific, Marlborough, MA, USA). Among DES cases, Eluvia (paclitaxel with a poly[glycolide-co-lactide] fluoropolymer, 120-µm strut thickness, Boston Scientific) was used in more than 60% of all cases. The temporal patterns of devices used during enrollment are illustrated in Supplemental Figure S1. A higher proportion of patients with infrapopliteal artery disease was observed in the CLTI group (CLTI: 48.6%; IC: 18.4%), more frequently involving the anterior tibial artery (ATA). The prevalence of calcified lesions was higher in the CLTI group (60.7%) than in the IC group (52.5%). However, the prevalence of total occlusion was similar in both groups (CLTI, 48.5%; IC, 48.6%). The patients in the CLTI group underwent subintimal tracking more frequently than those in the IC group (CLTI, 19.4%; IC, 14.3%). Patients in the IC group underwent plaque modification strategies, such as atherectomy (CLTI, 10.5%; IC, 11.6%), directional atherectomy, and anti-restenotic therapy (DAART; CLTI, 8.7%; IC, 10.7%). Plaque modification strategies, including atherectomy (CLTI, 10.5%; IC, 11.6%), directional atherectomy, and anti-restenotic therapy (DAART; CLTI, 8.7%; IC, 10.7%), were more frequently performed in the IC group.

Table 3 summarizes the procedural outcomes and medications administered at discharge. The initial procedural success rate, defined as residual stenosis of ≤30%, was identical in both groups (CLTI: 96.6%, IC: 96.6%). However, procedural complications, including bleeding and vascular rupture, occurred more frequently in the CLTI group (6.2%) than in the IC group (2.7%).

### 3.2. Clinical Outcomes

Crude analysis (Table 4) revealed that CLTI was associated with a higher risk of adverse outcomes than IC. Patients with CLTI were at a greater risk of undergoing target limb amputation or clinically driven TER (hazard ratio [HR], 1.762; 95% confidence interval [CI], 1.385–2.242; *p* < 0.001). Additionally, CLTI was associated with an increased risk of loss of clinical patency, MALEs, and all-cause mortality (HR, 1.715; 95% CI, 1.428–2.059; *p* < 0.001). The risk of all-cause mortality was substantially higher in patients with CLTI (HR, 4.067; 95% CI, 2.794–5.920; *p* < 0.001). **The risk of symptom aggravation was also higher in the CLTI group (HR 1.679, 95% CI [1.399–2.016], *p* < 0.001). Among patients who experienced symptom aggravation, only 11.8% in the CLTI group and 15.0% in the IC group subsequently underwent CD-TER.** Furthermore, CLTI was associated with an increased risk of minor (HR, 15.12; 95% CI, 6.919–33.06; *p* < 0.001) and major (HR, 14.01; 95% CI, 3.266–60.13; *p* < 0.001) lower limb amputations. However, the risk of repeat procedures was similar in both groups (HR, 1.177; 95% CI, 0.894–1.549; *p* = 0.259). After IPTW adjustment, the higher incidence of adverse events in the CLTI group remained significant, whereas the risk of repeat procedures did not differ between the groups (HR, 1.014; 95% CI, 0.833–1.234; *p* = 0.920). **These findings remained consistent in the time-stratified sensitivity analysis (2006–2014 and 2014–2021), indicating that the association between CLTI and adverse outcomes was stable across different treatment eras. Amputation-free survival curves stratified by clinical presentation and Rutherford classification are presented in** Supplementary Figure S2.

### 3.3. Subgroup Analysis: Treatment Strategy and Clinical Outcomes

**Figure 3:**
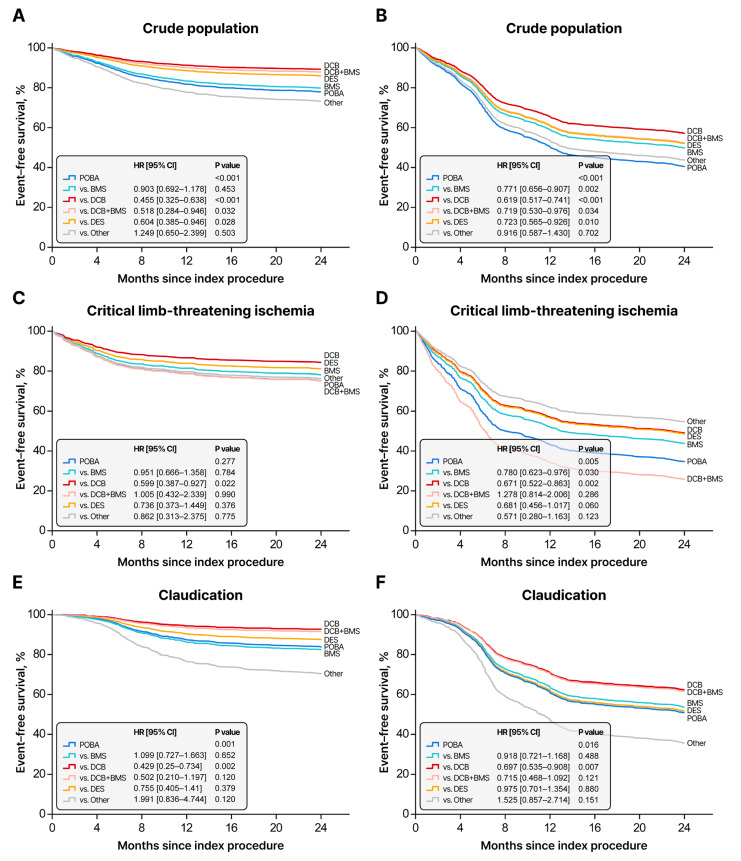
Two-year clinical outcomes according to the treatment strategy; Kaplan–Meier curves for event-free survival based on treatment strategy using the Cox proportional hazards model with inverse probability of treatment weighting: (**A**,**C**,**E**) represent the primary endpoint: target limb amputation or clinically driven target extremity revascularization in the crude population (**A**), critical limb ischemia (**C**), and claudication (**E**) groups. (**B**,**D**,**F**) depict the secondary endpoint: loss of clinical patency, major adverse limb events (MALEs), or all-cause mortality in the crude population (**B**), critical limb ischemia (**D**), and claudication (**F**) groups. Treatment strategies include POBA, BMS, DES, DCB, and DCB + BMS. POBA, plain old balloon angioplasty; BMS, bare-metal stent; DCB, drug-coated balloon; DES, drug-eluting stent.

DCB was associated with a lower risk of target limb amputation or clinically driven TER (hazard ratio [HR], 0.455; 95% confidence interval [CI] 0.325–0.638; *p* < 0.001) in the crude population. **The HR of the DCB-only group was 0.443 (95% CI, 0.317–0.621), whereas that of the combination of DCB with a BMS bail-out strategy was 0.518 (95% CI, 0.284–0.946). These findings show that provisional BMS implantation following DCB slightly attenuates the effect size of DCB; however, it does not account for the overall benefit observed in the total DCB group.** EVT with DES showed a lower risk of target limb amputation or clinically driven TER than POBA (HR, 0.604; 95% CI, 0.358–0.946; *p* = 0.028), although the benefit was less pronounced than that of DCB. (Figure 3). **Baseline characteristics of each treatment strategy subgroup are shown in** Supplementary Tables S3 and S4.

## 4. Discussion

The major findings of the present study are as follows: (1) The risk of target limb amputation or CD-TER was approximately 1.3-fold higher in the CLTI group compared to the IC group; (2) the risks of all-cause mortality, target limb amputation, MALEs, and loss of clinical patency in the CLTI group were 2.4, 7.8, 1.5, and 1.3 times higher, respectively, compared to the IC group at the 2-year follow-up; (3) no significant difference were observed between the CLTI and IC groups in terms of the rates of repeat PTA; and (4) a potential benefit of DCB in reducing the primary and secondary outcomes was observed in both groups.

The Global Burden of Disease, Injuries, and Risk Factors Study 2017 (GBD 2017) revealed that peripheral vascular disease accounted for 25.6% of the global burden of cardiovascular diseases and 1.7% of the overall global disease burden [14]. A retrospective analysis of the U.S. healthcare database (2003–2008) reported PAD prevalence and incidence rates of 10.7% and 2.4%, respectively [15]. A diagnosis of PAD poses a substantial economic burden on communities [16]. Despite the increasing prevalence of PAD in Korea due to the aging population, Asian populations remain underrepresented in PAD studies, leaving the current prevalence unclear. In recent Korean national health insurance data, a 20–25% annual increase in procedures was observed between 2008 and 2012, with EVT accounting for 80–95% of lower extremity arterial procedures [17,18]. Thus, PAD and its complications pose a growing burden on global morbidity and the economy.

Patients with CLTI typically present with more severe comorbidities than those with IC [2,19]. CLTI, often accompanied by diabetes mellitus and multiple comorbidities, such as metabolic disorders, renal failure, and prior cardiac disease, is characterized by severe inflammation and tissue necrosis, which limit treatment options [20]. In contrast, although claudication causes pain and reduced mobility due to decreased blood flow, it does not result in tissue loss and can be managed conservatively. The differences in the natural course and outcomes between CLTI and claudication following EVT have not been studied in real-world clinical settings, despite the poorer prognosis of CLTI.

The strengths of the K-VIS ELLA study lie in its large-scale real-world registry data, prospective enrollment, and long-term follow-up. Unlike previous studies that excluded Rutherford class 6 patients, this study included all patients, encompassing Rutherford class 6. Notably, most patients in the other studies were classified as Rutherford class 4. In the IN.PACT post hoc analysis, all-cause mortality (37.4% vs. 17.4%) and major limb amputation rates (6.8% vs. 1.1%) were higher in the CLTI group, whereas the 5-year freedom from CD-TER rate was lower (60.7% vs. 70.5%) [19]. However, the limited number of patients with CLTI (n = 156) compared with IC patients (n = 1246) prevented us from drawing definite conclusions. Another single-center study reported that the overall risk of reintervention at 3.7 years was similar between the groups (CLTI: 53.8%, IC: 60.2%), consistent with our findings [12]. However, this was a retrospective study, and the Rutherford classification was not available. In the present study, the rates of repeat procedures among patients with CLTI and IC were 13.1% and 11.4% (HR: 1.177, 95% CI: 0.894–1.549), respectively. Thus, existing evidence, including our findings, suggests that despite worse clinical outcomes in patients with CLTI, the need for repeat revascularization may not significantly differ from that in patients with IC.

These paradoxical findings of similar repeat revascularization rates despite markedly poorer outcomes in patients with CLTI raise important questions regarding the current treatment patterns. These findings may indicate that patients with CLTI do not receive sufficiently aggressive and timely interventions despite a higher risk of adverse events. Although various treatment options and dedicated guidelines are available for CLTI, late presentation and delayed management frequently increase the risk of amputation. Current guidelines emphasize the importance of diagnostic imaging in revascularization strategies in patients with CLTI [5]. However, the timing and aggressiveness of the treatment approaches in real-world practice are heterogeneous. Patients often undergo major amputations without proper angiographic evaluation [21]. As shown in Supplementary Table S5, the proportion of patients undergoing repeat PTA following symptom aggravation was similar between CLTI and IC, yet 11.7% of CLTI patients proceeded directly to amputation without any repeat EVT. This gap suggests that, despite having a substantially higher disease burden, a subset of patients with CLTI may not receive sufficiently timely reintervention. Below-the-knee procedures are technically challenging, time-consuming, and associated with poorer-than-usual long-term patency rates in this population. Thus, systematic evaluation of the vascular status and prompt revascularization are key elements affecting the management of CLTI. Our findings suggest that more intensive monitoring and structured follow-up protocols are essential for patients with CLTI, as similar rates of repeat revascularization, despite a higher amputation risk, may indicate suboptimal surveillance.

The ‘leave nothing behind’ strategy, using a drug-eluting device, is preferred for the management of extensive FPA disease. The findings of the present study indicated that compared with other treatment strategies, EVT with DCB was associated with a lower risk of target limb amputation or CD-TER, loss of clinical patency, MALEs, and all-cause mortality. Randomized clinical trials have confirmed the superiority of DCB over POBA in terms of its ability to reduce the incidence of binary restenosis and target lesion revascularization (TLR) in patients with relatively short FPA lesions. Moreover, prospective registry studies have indicated the efficacy of DCB in the management of more complex FPA lesions, including diffuse lesions and in-stent restenosis [22,23,24,25,26,27]. Compared with POBA, EVT with DCB significantly improves primary patency [6,7]. A previous study comparing EVT with DCB and DES revealed that patency rates were similar in both groups [8,9]. In contrast, the TLR rate associated with EVT with BMS was higher than that associated with POBA, possibly because of the increased risk of restenosis from the metallic scaffold without antiproliferative drug effects. The findings of the present study further support these observations, demonstrating that EVT with DCB is associated with a lower risk of adverse events than the other treatment strategies.

In this cohort, we observed that DCB and DES have gradually become the forefront devices in the management of FPA disease, with their use increasingly marked since the mid-2010s. Consistent with this shift in real-world practice, there was a progressive decline in POBA and BMS during the study period. Although EVT technology has evolved substantially over the 15-year study period, our sensitivity analysis stratified by enrollment era (2006–2014 and 2014–2021) showed that comparative clinical outcomes between patients with CLTI and IC remained consistent across periods. This suggests that advancements in device technology did not modify the fundamental differences in prognosis between CLTI and IC, indicating that disease biology rather than EVT remains the primary driver of outcomes. Comprehensive management, including optimal medical therapy, aggressive risk-factor control, and rehabilitation, may have an additional role in improving the prognosis of patients with CLTI in addition to EVT.

The findings of this study should be interpreted within the context of certain limitations. First, given the retrospective nature of this multicenter registry study, the potential for selection and information biases could not be entirely excluded. Although the IPTW analysis was conducted to mitigate these biases, unmeasured confounding factors may have influenced the outcomes. Second, the absence of the wound, ischemia, and foot infection classification limits the detailed evaluation of severity. Third, variability in device selection and procedural strategies may exist due to reliance on individual physician discretion. Fourth, routine imaging and functional assessments used to evaluate vessel patency after the procedure were not uniform. Fifth, in the subgroup analyses of treatment strategies and clinical outcomes, the choice of device was left to the operator’s discretion. Therefore, these findings should be interpreted with caution, as differences in lesion characteristics, along with indication bias, may have influenced the selection of devices and consequently the observed outcomes. Sixth, in cases of technical failure or peri-procedural complications after EVT, such as flow-limiting dissection, vascular rupture, or access-site events, detailed information on subsequent management strategies was not systematically captured in the registry, and post-complication treatments may have varied among operators. Our dataset does not provide information on whether these cases required surgical conversion or other adjunctive procedures. Thus, detailed therapeutic approaches to failed EVT cannot be fully assessed. Finally, variations in postoperative medical management, including antiplatelet therapy duration and medication adherence across participating centers, might represent additional unaccounted confounders. Especially, longitudinal information on drug adherence was not available. Although dual antiplatelet therapy was recommended for more than 6 months following EVT, actual post-discharge medical treatment was at the discretion of the physicians.

## 5. Conclusions

This real-world registry study of patients undergoing EVT for FPA disease demonstrated that the clinical outcomes of patients with CLTI were worse than those of patients with IC. However, this difference in outcomes did not extend to the risk of repeat PTA, which was similar in both groups at the 2-year follow-up. The present study revealed that compared to POBA, the use of DCB was associated with superior outcomes in both groups.

## Figures and Tables

**Table 1:** Baseline characteristics and laboratory findings.

	Crude Population			IPTW Population		
Variables Mean ± SD or n (%)	CLTI (n = 812)	Claudication (n = 1112)	SMD	CLTI (n = 1915)	Claudication (n = 2010)	SMD
Sex, male	618 (76.1)	953 (85.7)	0.011	1565 (81.7)	1642 (81.6)	0.000
Age, years	70.3 ± 10.7	69.0 ± 10.3	−0.013	69.1 ± 11.1	70.4 ± 10.9	0.011
Body mass index, kg/m^2^	22.6 ± 3.5	23.6 ± 3.2	0.029	23.3 ± 3.7	23.1 ± 3.2	−0.007
Patient risks						
Hypertension	601 (74.0)	855 (76.8)	0.003	1441 (75.2)	1464 (72.8)	−0.003
Diabetes mellitus	581 (71.5)	672 (60.4)	−0.014	1266 (66.1)	1302 (64.7)	−0.002
Dyslipidemia	422 (51.9)	689 (61.9)	0.013	1086 (56.7)	1095 (54.4)	−0.003
Chronic kidney disease	288 (35.4)	210 (18.8)	−0.032	507 (26.4)	538 (26.7)	0.001
eGFR-MDRD	60 ± 39	69 ± 33	0.026	64 ± 36	65 ± 37	0.002
ESRD	188 (23.1)	101 (9)	−0.035	295 (15.4)	272 (13.5)	−0.005
COPD	32 (3.9)	41 (3.6)	−0.001	86 (4.4)	81 (4.0)	−0.002
Congestive heart failure	54 (6.6)	37 (3.3)	−0.015	90 (4.6)	84 (4.1)	−0.002
Coronary artery disease	370 (45.5)	606 (54.4)	0.013	973 (50.8)	975 (48.5)	−0.003
Prior MI	60 (7.3)	92 (8.2)	0.003	146 (7.6)	154 (7.6)	0.000
Prior PCI	198 (24.3)	361 (32.4)	0.015	580 (30.2)	573 (28.5)	−0.003
Prior CABG	72 (8.8)	97 (8.7)	0.000	154 (8.0)	156 (7.7)	−0.001
Stroke	166 (20.4)	177 (15.9)	−0.011	346 (18.0)	336 (16.7)	−0.003
Prior PTA	287 (35.3)	351 (31.5)	−0.007	646 (33.7)	650 (32.3)	−0.002
Smoking	195 (24.0)	331 (29.7)	0.011	559 (29.1)	517 (25.7)	−0.007
Laboratory findings						
Glucose levels, mg/dL	152 ± 82	145 ± 74	−0.010	148 ± 77	150 ± 81	0.003
Glycated hemoglobin, %	7.7 ± 5.7	7.5 ± 5.7	−0.004	7.5 ± 4.3	7.4 ± 4.6	−0.002
Creatinine levels, mg/dL	2.26 ± 2.53	1.61 ± 2.05	−0.028	1.93 ± 2.25	1.88 ± 2.28	−0.002
Hemoglobin levels, mg/dL	11.4 ± 2.2	12.6 ± 3.5	0.041	12.1 ± 2.4	11.9 ± 3.1	−0.006
Total cholesterol levels, mg/dL	141 ± 40	143 ± 39	0.006	145 ± 41	143 ± 39	−0.006
Triglyceride levels, mg/dL	121 ± 76	139 ± 95	0.021	144 ± 129	130 ± 85	−0.012
HDL levels, mg/dL	37 ± 11	41 ± 11	0.031	39 ± 12	39 ± 11	−0.003
LDL levels, mg/dL	81 ± 32	79 ± 33	−0.005	81 ± 32	80 ± 33	−0.004

CLTI, critical limb-threatening ischemia; SMD, standardized mean difference; eGFR, estimated glomerular filtration rate; MDRD, modification of diet in renal disease; ESRD, end-stage renal disease; COPD, chronic obstructive pulmonary disease; MI, myocardial infarction; PCI, percutaneous coronary intervention; CABG, coronary artery bypass graft.

**Table 2:** Angiographic and procedural characteristics.

	Crude Population			IPTW Population		
Variables Mean ± SD or n (%)	CLTI (n = 812)	Claudication (n = 1112)	SMD	CLTI (n = 1915)	Claudication (n = 2010)	SMD
Angiographic and procedural characteristics						
Limb side, Right	395 (48.6)	543 (48.8)	0.000	956 (49.9)	983 (48.9)	−0.001
Procedural approach						
Ipsilateral	311 (38.3)	260 (23.3)	−0.027	575 (30.0)	589 (29.3)	−0.001
Contralateral	525 (64.6)	888 (79.8)	0.018	1410 (73.6)	1475 (73.3)	0.000
Lesion location						
Distal aorta	108 (13.3)	162 (14.5)	0.003	271 (14.1)	323 (16.0)	0.005
Common iliac artery	108 (13.3)	167 (15.0)	0.005	271 (14.1)	328 (16.3)	0.006
Common femoral artery	25 (3.0)	45 (4.0)	0.005	62 (3.2)	61 (3.0)	−0.001
Superficial femoral artery	812 (100.0)	1112 (100.0)	-	1915 (100.0)	2010 (100.0)	-
Infra-popliteal artery	395 (48.6)	205 (18.4)	−0.052	594 (31.0)	670 (33.3)	0.004
Anterior tibial artery	227 (27.9)	74 (6.6)	−0.051	331 (17.2)	342 (17.0)	−0.001
Posterior tibial artery	157 (19.3)	44 (3.9)	−0.045	203 (10.6)	308 (15.3)	0.013
Peroneal artery	110 (13.5)	39 (3.5)	−0.034	148 (7.7)	168 (8.3)	0.002
Total occlusion lesion	394 (48.5)	541 (48.6)	0.000	957 (49.9)	1000 (49.7)	0.000
Calcified lesion	493 (60.7)	584 (52.5)	−0.011	1020 (53.2)	1124 (55.9)	0.004
**Moderate/Severe calcification**	**260 (32.0)**	**312 (28.1)**	**−0.087**	**547 (28.6)**	**669 (33.3)**	**0.102**
**TASC-II (A/B)**	**329 (40.5)**	**497 (44.7)**	**0.085**	**776 (40.5)**	**824 (41.0)**	**0.010**
**TASC-II (C/D)**	**483 (59.5)**	**615 (55.3)**	**−0.085**	**1139 (59.5)**	**1186 (59.0)**	**−0.010**
**Lesion length ≥ 150 mm**	**461 (56.8)**	**532 (48.1)**	**−0.176**	**1076 (56.3)**	**1112 (55.6)**	**−0.014**
**Distal runoff vessels**	**2.39 (0.85)**	**2.86 (0.45)**	**0.069**	**2.59 (0.80)**	**2.64 (0.70)**	**0.007**
lesion stenosis, %	89.7 ± 13.1	91.1 ± 11.6	0.011	90.6 ± 12.4	90.7 ± 12.3	0.001
Lesion diameter, mm (max)	6.08 ± 0.9	6.24 ± 0.8	0.019	6.14 ± 0.87	6.18 ± 0.79	0.004
Total lesion length, mm	175 ± 105	163 ± 110	−0.011	173 ± 104	173 ± 106	0.000
Sub-intimal approach	158 (19.4)	160 (14.3)	−0.012	322 (16.8)	306 (15.2)	−0.004
Atherectomy	86 (10.5)	130 (11.6)	0.003	224 (11.6)	288 (14.3)	0.007
DAART	71 (8.7)	120 (10.7)	0.007	194 (10.1)	249 (12.3)	0.007
Lesion treatment						
POBA only	231 (28.4)	215 (19.3)	−0.019	448 (23.3)	451 (22.4)	−0.002
BMS only	262 (32.2)	382 (34.3)	0.004	618 (32.2)	615 (30.5)	−0.003
DCB only	203 (25.0)	304 (27.3)	0.005	508 (26.5)	611 (30.3)	0.007
DCB + BMS	31 (3.8)	71 (6.3)	0.011	115 (6.0)	99 (4.9)	−0.005
DES only	61 (7.5)	115 (10.3)	0.009	179 (9.3)	172 (8.5)	−0.003
**Type of DCB**						
**IN.PACT Admiral**	**161 (73.9)**	**261 (74.6)**	**0.089**	**440 (68.8)**	**472 (73.8)**	**0.108**
**Lutonix**	**41 (18.8)**	**38 (10.9)**	**−0.081**	**108 (16.9)**	**97 (15.2)**	**−0.045**
**Ranger**	**12 (5.5)**	**42 (12.0)**	**0.139**	**92 (14.4)**	**71 (11.1)**	**−0.103**

CLTI, critical limb-threatening ischemia; SMD, standardized mean difference; DAART, directional atherectomy and antirestenotic therapy; POBA, plain old balloon angioplasty; BMS, bare metal stent; DCB, drug-coated balloon; DES, drug-eluting stent; **TASC-II = Trans-Atlantic Inter-Society Consensus II.**

**Table 3:** Procedural outcomes and medications at discharge.

	Crude Population			IPTW Population		
Variables Mean ± SD or n (%)	CLTI (n = 812)	Claudication (n = 1112)	*p*-Value	CLTI (n = 1915)	Claudication (n = 2010)	*p*-Value
Procedural outcomes						
Residual stenosis, >30%	17 (2.0)	23 (2.0)	0.969	27 (1.4)	33 (1.6)	0.554
Technical success	785 (96.6)	1075 (96.6)	0.998	1869 (97.5)	1958 (97.4)	0.710
**Ankle brachial index (ABI)**						
ABI before PTA	0.59 ± 0.26	0.62 ± 0.18	0.019	0.59 ± 0.23	0.60 ± 0.18	0.155
ABI after PTA	0.87 ± 0.20	0.89 ± 0.17	0.028	0.87 ± 0.20	0.90 ± 0.16	0.001
**Procedural complications**						
Any complications	51 (6.2)	31 (2.7)	<0.001	101 (5.2)	62 (3.0)	0.001
Bleeding	33 (4.0)	20 (1.7)	0.003	60 (3.1)	41 (2.0)	0.030
Vascular rupture	15 (1.8)	8 (0.7)	0.025	33 (1.7)	15 (0.7)	0.005
Distal embolization	6 (0.7)	3 (0.2)	0.180	13 (0.6)	5 (0.2)	0.046
**Discharge medications**						
Aspirin	612 (75.3)	901 (81.0)	0.003	1517 (79.2)	1549 (77.0)	0.103
P2Y12 inhibitors	616 (75.8)	964 (86.6)	<0.001	1539 (80.3)	1718 (85.4)	<0.001
Cilostazol	212 (26.1)	365 (32.8)	0.001	477 (24.9)	612 (30.4)	<0.001
Beraprost	95 (11.6)	62 (5.5)	<0.001	170 (8.8)	124 (6.1)	0.001
Triflusal	2 (0.2)	16 (1.4)	0.007	8 (0.4)	27 (1.3)	0.002
Sarpogrelate	3 (0.3)	3 (0.2)	0.702	6 (0.3)	4 (0.1)	0.539
Anticoagulants	81 (9.9)	97 (8.7)	0.349	209 (10.9)	159 (7.9)	0.001
Warfarin	65 (8.0)	73 (6.5)	0.227	155 (8.0)	115 (5.7)	0.003
NOAC	16 (1.9)	24 (2.1)	0.776	54 (2.8)	44 (2.1)	0.206
RAS inhibitors	332 (40.8)	569 (51.1)	<0.001	832 (43.4)	898 (44.6)	0.438
ACE inhibitors	71 (8.7)	121 (10.8)	0.122	190 (9.9)	195 (9.7)	0.817
ARBs	267 (32.8)	456 (41)	<0.001	657 (34.3)	713 (35.4)	0.444
β-blockers	276 (33.9)	393 (35.3)	0.539	682 (35.6)	723 (35.9)	0.816
Calcium channel blockers	275 (33.8)	404 (36.3)	0.264	663 (34.6)	730 (36.3)	0.272
Diuretics	157 (19.3)	182 (16.3)	0.091	358 (18.6)	349 (17.3)	0.278
Statins	536 (66.0)	869 (78.1)	<0.001	1399 (73.0)	1414 (70.3)	0.060

CLTI, critical limb-threatening ischemia; PTA, percutaneous transluminal angioplasty; NOAC, new oral anticoagulants; RAS, renin–angiotensin–aldosterone system; ACE, angiotensin-converting enzyme; ARB, angiotensin receptor blocker.

**Table 4:** Clinical outcomes and hazard ratio of critical limb ischemia up to 2 years.

Outcomes Mean ± SD or n (%)	CLTI	Claudication	*p*-Value	HR [95% CI]	*p*-Value
Crude population	(n = 812)	(n = 1112)			
Primary endpoint	174 (21.4)	149 (13.3)	<0.001	1.762 [1.385–2.242]	<0.001
Any amputation	71 (8.7)	7 (0.6)	<0.001	15.12 [6.919–33.06]	<0.001
Clinically driven TER	96 (11.8)	116 (10.4)	0.336	1.151 [0.864–1.533]	0.339
Secondary endpoint	471 (58.0)	496 (44.6)	<0.001	1.715 [1.428–2.059]	<0.001
Death	107 (13.1)	40 (3.5)	<0.001	4.067 [2.794–5.920]	<0.001
Cardiac death	47 (5.7)	16 (1.4)	<0.001	4.208 [2.368–7.477]	<0.001
Non-cardiac death	60 (7.3)	24 (2.1)	<0.001	3.617 [2.232–5.859]	<0.001
Major amputation	20 (2.4)	2 (0.1)	<0.001	14.01 [3.266–60.13]	<0.001
Repeated PTA	107 (13.1)	127 (11.4)	0.244	1.177 [0.894–1.549]	0.259
Non-TER	12 (1.4)	11 (0.9)	0.330	1.501 [0.659–3.419]	0.397
MALEs	161 (19.8)	132 (11.8)	<0.001	1.836 [1.429–2.358]	<0.001
Symptom aggravation	453 (55.7)	477 (42.8)	<0.001	1.679 [1.399–2.016]	<0.001
Clinical patency	354 (43.5)	628 (56.4)	<0.001	1.678 [1.398–2.014]	<0.001
IPTW population	(n = 1915)	(n = 2010)			
Primary endpoint	337 (17.5)	281 (13.9)	0.002	1.314 [1.105–1.561]	0.002
Any amputation	120 (6.2)	17 (0.8)	<0.001	7.837 [4.697–13.076]	<0.001
Clinically driven TER	197 (10.2)	211 (10.4)	0.829	0.977 [0.796–1.200]	0.834
Secondary endpoint	994 (51.9)	907 (45.1)	<0.001	1.312 [1.157–1.488]	<0.001
Death	184 (9.6)	85 (4.2)	<0.001	2.407 [1.846–3.138]	<0.001
Cardiac death	75 (3.9)	33 (1.6)	<0.001	2.441 [1.613–3.695]	<0.001
Non-cardiac death	109 (5.6)	51 (2.5)	<0.001	2.318 [1.652–3.252]	<0.001
Major amputation	34 (1.7)	8 (0.3)	<0.001	4.523 [2.088–9.796]	<0.001
Repeated PTA	221 (11.5)	229 (11.3)	0.885	1.014 [0.833–1.234]	0.920
Non-TER	26 (1.3)	18 (0.8)	0.169	1.523 [0.832–2.787]	0.176
MALEs	318 (16.6)	243 (12.0)	<0.001	1.447 [1.209–1.733]	<0.001
Symptom aggravation	956 (49.9)	877 (43.6)	<0.001	1.287 [1.135–1.460]	<0.001
Clinical patency	944 (49.2)	1120 (55.7)	<0.001	1.294 [1.141–1.467]	<0.001

CLTI, critical limb-threatening ischemia; HR, hazard ratio; CI, confidence interval; PTA, percutaneous transluminal angioplasty; TER, target extremity revascularization; MALE, major adverse limb event.

## Data Availability

The data supporting the findings of this study are available from the corresponding author upon request.

## Supplementary Materials

The following supporting information can be downloaded at: https://www.mdpi.com/article/10.3390/jcm14248919/s1, Figure S1: Temporal trend of the device used (2006–2021); Figure S2: Two-year amputation-free survival according to clinical presentation and Rutherford classification; Table S1: List of devices; Table S2: Clinical outcomes after EVT stratified by treatment era (2006–2014 and 2014–2021); Table S3: Baseline characteristics and laboratory findings stratified by treatment strategy; Table S4: Angiographic and procedural characteristics stratified by treatment strategy; Table S5: Patients who experienced worsening symptom after EVT.

## Abbreviations

The following abbreviations are used in this manuscript:

CLTI	critical limb-threatening ischemia
DCB	drug-coated balloon
DES	drug-eluting stent
FPA	femoropopliteal artery
IC	intermittent claudication
IPTW	inverse probability weighting
POBA	plain old balloon angioplasty
PTA	percutaneous transluminal angioplasty
