# Supplementary material for: Two-Year Clinical Outcomes of Critical Limb-Threatening Ischemia Versus Claudication After Femoropopliteal Endovascular Therapy: An Analysis from K-VIS ELLA Registry
Source: J Clin Med. 2025 Dec 17;14(24):8919. doi: 10.3390/jcm14248919 (PMC12734144; doi:10.3390/jcm14248919)
Supplement: Supplementary file 1 [file jcm-14-08919-s001.zip › Supplement Figures.pptx]

## Slide 1
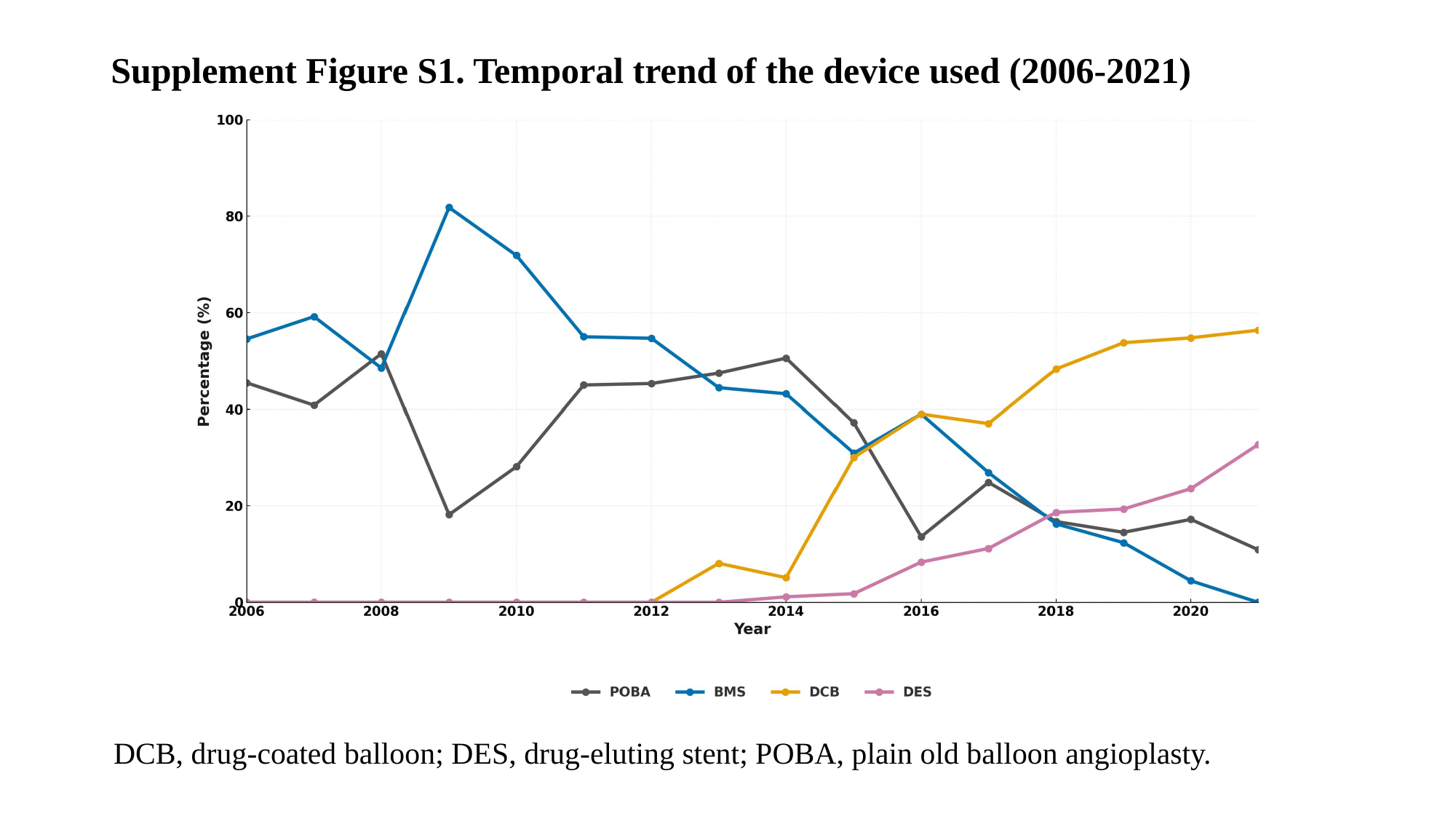

# Supplement Figure S1. Temporal trend of the device used (2006-2021)
DCB, drug-coated balloon; DES, drug-eluting stent; POBA, plain old balloon angioplasty.

## Slide 2
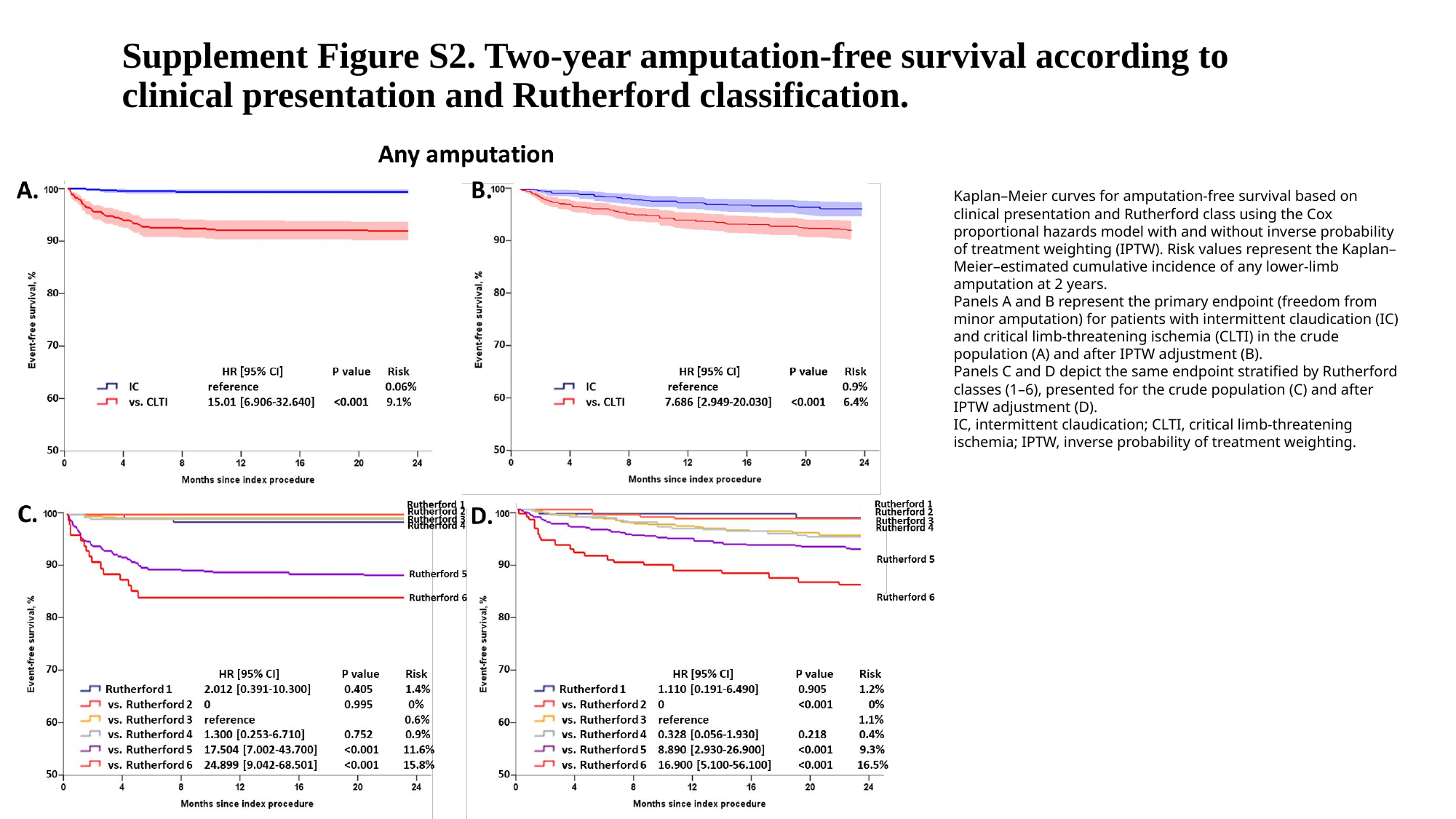

Supplement Figure S2. Two-year amputation-free survival according to clinical presentation and Rutherford classification.
Kaplan–Meier curves for amputation-free survival based on clinical presentation and Rutherford class using the Cox proportional hazards model with and without inverse probability of treatment weighting (IPTW). Risk values represent the Kaplan–Meier–estimated cumulative incidence of any lower-limb amputation at 2 years.
Panels A and B represent the primary endpoint (freedom from minor amputation) for patients with intermittent claudication (IC) and critical limb-threatening ischemia (CLTI) in the crude population (A) and after IPTW adjustment (B).
Panels C and D depict the same endpoint stratified by Rutherford classes (1–6), presented for the crude population (C) and after IPTW adjustment (D).
IC, intermittent claudication; CLTI, critical limb-threatening ischemia; IPTW, inverse probability of treatment weighting.
