# Supplementary material for: Two-Year Clinical Outcomes of Critical Limb-Threatening Ischemia Versus Claudication After Femoropopliteal Endovascular Therapy: An Analysis from K-VIS ELLA Registry
Source: J Clin Med. 2025 Dec 17;14(24):8919. doi: 10.3390/jcm14248919 (PMC12734144; doi:10.3390/jcm14248919)
Supplement: Supplementary file 1 [file jcm-14-08919-s001.zip › Supplement Tables.pdf]

Supplemental Table S1. List of devices

Bare-metal stent (N=695)			
**Product**	**Total**	**CLTI**	**IC**
**Smart**	264 (38.0%)	89 (30.6%)	175 (39.6%)
**Absolute Pro**	130 (18.7%)	62 (21.3%)	68 (15.4%)
**Supera**	91 (13.1%)	34 (11.7%)	57 (12.9%)
**Complete SE**	84 (12.1%)	39 (13.4%)	45 (10.2%)
**Others***	126 (18.1%)	67 (23.0%)	97 (21.9%)
**Total BMS devices**	695 (100%)	291 (100%)	442 (100%)
Drug-eluting stent (N=187)			
**Product**	**Total**	**CLTI**	**IC**
**Eluvia**	120 (64.2%)	39 (57.4%)	81 (68.1%)
**Zilver PTX**	67 (35.8%)	29 (42.6%)	38 (31.9%)
**Total DES devices**	187 (100%)	68 (100%)	119 (100%)
Drug-coated balloon (N=568)			
**Product**	**Total**	**CLTI**	**IC**
**IN.PACT Admiral**	422 (74.3%)	161 (73.9%)	261 (74.6%)
**Lutonix**	79 (13.9%)	41 (18.8%)	38 (10.9%)
**Ranger**	54 (9.5%)	12 (5.5%)	42 (12.0%)
**Others†**	13 (2.3%)	4 (1.8%)	9 (2.6%)
**Total DCB devices**	568 (100%)	218 (100%)	350 (100%)

CLTI, critical limb-threatening ischemia; IC, intermittent claudication.
*Others include Innova, Protege, Misago, Epic, Life, Omnilink, Palmaz, Maris, Express LD, Pulsar, Scuba etc.
†Others include Passeo and SeQuent Please (low-frequency use).

Supplemental Table S2. Clinical outcomes after EVT stratified by treatment era (2006-2014 and 2014-2021)

Outcome (2006-2014)	Group	N	Event	Crude		IPTW	
				HR (95% CI)	P-value	HR (95% CI)	P-value
**Primary endpoint**	**IC**	398	65	1.000 (reference)		1.00 (reference)	
	**CLTI**	271	70	1.850 (1.320-2.593)	<0.001	1.687 (1.181–2.41)	<0.001
**Secondary endpoint**	**IC**	398	189	1.000 (reference)		1.00 (reference)	
	**CLTI**	271	160	1.526 (1.236-1.884)	<0.001	1.394 (1.113–1.746)	0.004
**All-cause death**	**IC**	398	18	1.000 (reference)		1.00 (reference)	
	**CLTI**	271	34	2.922 (1.650-5.173)	<0.001	2.207 (1.181–4.124)	0.013
**Repeated PTA**	**IC**	398	54	1.00 (reference)		1.00 (reference)	
	**CLTI**	271	44	1.316 (0.884-1.959)	0.177	1.230 (0.804–1.882)	0.339
**Total amputation**	**IC**	398	3	1.00 (reference)		1.00 (reference)	
	**CLTI**	271	27	14.152 (4.293-46.654)	<0.001	23.598 (5.570–99.986)	<0.001
**Outcome (2014-2021)**							
**Primary endpoint**	**IC**	714	84	1.000 (reference)		1.00 (reference)	
	**CLTI**	541	104	1.894 (1.423-2.529)	<0.001	1.679 (1.235–2.281)	<0.001
**Secondary endpoint**	**IC**	714	307	1.000 (reference)		1.00 (reference)	
	**CLTI**	541	311	1.668 (1.424-1.953)	<0.001	1.547 (1.305–1.834)	<0.001
**All-cause death**	**IC**	714	22	1.000 (reference)		1.00 (reference)	
	**CLTI**	541	73	4.655 (7.499-2.890)	<0.001	3.806 (2.315–6.259)	<0.001
**Repeated PTA**	**IC**	714	73	1.00 (reference)		1.00 (reference)	
	**CLTI**	541	63	1.262 (0.901-1.768)	0.176	1.192 (0.829–1.714)	0.344

**Total amputation**	**IC**	714	4	1.00 (reference)		1.00 (reference)	
	**CLTI**	541	44	15.711 (5.645-43.731)	<0.001	12.426 (4.259–36.254)	<0.001

CI, confidence interval; CLTI, critical limb-threatening ischemia; HR, hazard ratio; IC, intermittent claudication; IPTW, inverse probability of treatment weighting; PTA, percutaneous transluminal angioplasty.

Supplement Table S3. Baseline characteristics and laboratory findings stratified by treatment strategy

Crude population

**Variables**<br>**mean ± SD or n (%)**	**POBA**<br>**(n=446)**	**BMS**<br>**(n=655)**	**DCB**<br>**(n=507)**	**DCB+BMS**<br>**(n=102)**	**DES**<br>**(n=176)**	**Others**<br>**(n=38)**	**SMD**
Sex, male	346 (77.6)	550 (84.0)	418 (82.4)	86 (84.3)	144 (81.8)	27 (71.1)	0.141
Age, year	68.22 (11.34)	70.42 (9.80)	69.52 (10.14)	70.28 (11.41)	70.38 (9.96)	66.61 (15.33)	0.146
Body mass index, kg/m2	22.95 (3.42)	23.02 (3.27)	23.54 (3.21)	23.34 (3.12)	23.76 (4.32)	22.75 (3.70)	0.133
Risks of patient							
CLTI	231 (51.8)	269 (41.1)	203 (40.0)	31 (30.4)	61 (34.7)	17 (44.7)	0.190
Hypertension	337 (75.6)	497 (75.9)	387 (76.3)	81 (79.4)	129 (73.3)	25 (65.8)	0.117
Diabetes mellitus	302 (67.7)	415 (63.4)	357 (70.4)	58 (56.9)	104 (59.1)	17 (44.7)	0.228
Dyslipidemia	246 (55.2)	331 (50.5)	334 (65.9)	58 (56.9)	120 (68.2)	22 (57.9)	0.167
Chronic kidney disease	137 (30.7)	134 (20.5)	152 (30.0)	20 (19.6)	47 (26.7)	8 (21.1)	0.139
ESRD	94 (21.1)	47 (7.2)	74 (14.6)	12 (11.8)	24 (13.6)	3 (7.9)	0.183
COPD	9 (2.0)	36 (5.5)	17 (3.4)	3 (2.9)	8 (4.5)	0 (0.0)	0.152
Congestive heart failure	23 (5.2)	31 (4.7)	23 (4.5)	6 (5.9)	5 (2.8)	3 (7.9)	0.09
Coronary artery disease	223 (50.0)	350 (53.4)	252 (49.7)	53 (52.0)	81 (46.0)	17 (44.7)	0.082
Prior MI	39 (8.7)	49 (7.5)	36 (7.1)	15 (14.7)	11 (6.2)	2 (5.3)	0.127
Prior PCI	127 (28.5)	188 (28.7)	145 (28.6)	33 (32.4)	53 (30.1)	13 (34.2)	0.060
Prior CABG	47 (10.5)	61 (9.3)	45 (8.9)	5 (4.9)	10 (5.7)	1 (2.6)	0.152

Stroke	81 (18.2)	123 (18.8)	94 (18.5)	14 (13.7)	27 (15.3)	4 (10.5)	0.110
Prior PTA	160 (35.9)	179 (27.3)	186 (36.7)	35 (34.3)	67 (38.1)	11 (28.9)	0.112
Smoking	94 (21.1)	207 (31.6)	125 (24.7)	34 (33.3)	55 (31.2)	11 (28.9)	0.127
Laboratory findings							
Glucose levels, mg/dL	155.41 (83.46)	140.71 (73.08)	155.91 (76.02)	137.71 (55.82)	144.12 (75.06)	149.75 (145.81)	0.116
Glycated hemoglobin, %	8.12 (7.63)	7.74 (6.93)	7.32 (2.93)	7.37 (1.56)	7.26 (1.61)	7.10 (1.51)	0.103
Creatinine levels, mg/dL	2.39 (2.78)	1.55 (1.83)	1.92 (2.28)	1.88 (2.42)	1.90 (2.24)	1.53 (2.21)	0.154
Hemoglobin levels, mg/dL	11.97 (5.22)	12.21 (2.15)	12.19 (2.14)	12.40 (2.24)	12.06 (1.86)	12.68 (1.85)	0.125
Total cholesterol levels, mg/dL	140.25 (37.09)	148.72 (39.45)	138.64 (40.68)	141.55 (40.05)	136.98 (38.87)	156.57 (48.14)	0.206
Triglyceride levels, mg/dL	126.66 (115.61)	135.47 (76.82)	133.79 (84.03)	133.27 (79.19)	125.91 (79.34)	143.09 (68.11)	0.091
HDL levels, mg/dL	40.09 (12.93)	38.80 (11.47)	41.74 (12.23)	38.29 (9.20)	41.18 (12.72)	38.62 (9.45)	0.153
LDL levels, mg/dL	79.14 (29.53)	85.23 (32.36)	77.33 (36.38)	79.44 (32.25)	74.50 (32.94)	79.40 (31.31)	0.123

IPTW population

**Variables**<br>**mean ± SD or n (%)**	**POBA**<br>**(n=899)**	**BMS**<br>**(n=1255)**	**DCB**<br>**(n=1119)**	**DCB+BMS**<br>**(n=213)**	**DES**<br>**(n=351)**	**Others**<br>**(n=88)**	**SMD**
Sex, male	696.3 (77.4)	1065.9 (84.9)	931.6 (83.3)	181.2 (84.9)	280.8 (80.0)	51.1 (58.1)	0.251
Age, year	67.44 (11.91)	70.94 (9.86)	70.79 (10.68)	67.48 (12.79)	70.23 (10.43)	68.93 (15.14)	0.162
Body mass index, kg/m2	22.75 (3.47)	23.04 (3.43)	23.48 (3.38)	24.06 (3.43)	23.97 (4.28)	22.61 (3.71)	0.209

Risks of patient							
CLTI	448.1 (49.8)	631.8 (50.3)	508.0 (45.4)	114.5 (53.7)	178.7 (50.9)	33.9 (38.6)	0.124
Hypertension	665.9 (74.1)	960.4 (76.5)	768.2 (68.7)	176.2 (82.6)	266.3 (75.8)	68.1 (77.5)	0.127
Diabetes mellitus	627.8 (69.8)	801.4 (63.9)	771.2 (69.0)	116.6 (54.6)	198.3 (56.5)	52.8 (60.1)	0.162
Dyslipidemia	495.3 (55.1)	633.0 (50.4)	649.9 (58.1)	111.9 (52.4)	245.5 (69.9)	45.2 (51.5)	0.165
Chronic kidney disease	278.1 (30.9)	254.5 (20.3)	359.5 (32.1)	35.4 (16.6)	99.4 (28.3)	17.4 (19.8)	0.186
ESRD	181.4 (20.2)	80.9 (6.4)	153.9 (13.8)	21.0 (9.8)	53.0 (15.1)	5.1 (5.8)	0.211
COPD	14.1 (1.6)	88.2 (7.0)	43.5 (3.9)	4.4 (2.1)	16.7 (4.8)	0.0 (0.0)	0.185
Congestive heart failure	40.6 (4.5)	60.8 (4.8)	43.3 (3.9)	8.6 (4.0)	14.6 (4.1)	6.1 (6.9)	0.054
Coronary artery disease	437.4 (48.6)	691.2 (55.1)	502.3 (44.9)	123.9 (58.1)	162.9 (46.4)	29.3 (33.4)	0.213
Prior MI	81.5 (9.1)	84.4 (6.7)	80.6 (7.2)	28.8 (13.5)	20.8 (5.9)	3.5 (4.0)	0.141
Prior PCI	260.1 (28.9)	375.6 (29.9)	299.6 (26.8)	88.2 (41.4)	107.0 (30.5)	22.5 (25.6)	0.130
Prior CABG	90.4 (10.0)	110.3 (8.8)	79.4 (7.1)	10.1 (4.8)	19.3 (5.5)	1.3 (1.4)	0.166
Stroke	153.7 (17.1)	242.1 (19.3)	178.7 (16.0)	34.6 (16.2)	48.0 (13.7)	25.1 (28.6)	0.143
Prior PTA	300.1 (33.4)	343.3 (27.4)	399.8 (35.7)	67.6 (31.7)	162.0 (46.1)	23.3 (26.5)	0.177
Smoking	201.4 (22.4)	389.8 (31.1)	266.0 (23.8)	80.7 (37.8)	114.5 (32.6)	23.5 (26.8)	0.159
Laboratory findings							
Glucose levels, mg/dL	152.34 (78.49)	140.19 (72.54)	164.38 (86.62)	137.73 (52.49)	139.47 (69.85)	139.89 (131.79)	0.142
Glycated hemoglobin, %	8.05 (6.17)	7.58 (5.32)	7.17 (2.36)	7.69 (1.70)	7.28 (1.63)	7.16 (1.50)	0.133
Creatinine levels, mg/dL	2.41 (2.80)	1.55 (1.79)	1.94 (2.18)	1.70 (2.21)	2.08 (2.46)	1.36 (2.03)	0.207

Hemoglobin levels, mg/dL	11.87 (4.18)	12.20 (2.18)	11.95 (2.40)	12.61 (2.26)	12.08 (1.97)	12.41 (1.95)	0.140
Total cholesterol levels, mg/dL	141.10 (37.64)	149.89 (40.29)	142.58 (41.55)	145.38 (42.47)	134.98 (39.32)	146.30 (42.46)	0.154
Triglyceride levels, mg/dL	144.64 (180.49)	136.95 (75.68)	135.34 (83.10)	146.22 (77.31)	120.52 (67.22)	139.65 (60.88)	0.126
HDL levels, mg/dL	39.84 (12.60)	38.99 (12.02)	40.90 (12.32)	39.64 (10.80)	40.14 (11.98)	38.40 (9.10)	0.095
LDL levels, mg/dL	77.96 (28.58)	84.29 (33.07)	82.60 (37.24)	80.81 (30.58)	72.62 (31.47)	77.41 (28.52)	0.161

Values are presented as mean ± standard deviation or number (percentage).

Treatment groups were categorized as follows: POBA, plain old balloon angioplasty; BMS, bare-metal stent; DCB, drug-coated balloon; DCB+BMS, provisional BMS implantation after DCB; DES, drug-eluting stent.

CLTI, critical limb-threatening ischemia; SMD, standardized mean difference; ESRD, end-stage renal disease; COPD, chronic obstructive pulmonary disease; MI, myocardial infarction; PCI, percutaneous coronary intervention; CABG, coronary artery bypass graft; PTA, percutaneous transluminal angioplasty; HDL, high-density lipoprotein; LDL, low-density lipoprotein.

Supplement Table S4. Angiographic and procedural characteristics stratified by treatment strategy

Variables<br>mean ± SD or n (%)	Crude population<br>POBA<br>(n=446)	BMS<br>(n=655)	DCB<br>(n=507)	DCB+BMS<br>(n=102)	DES<br>(n=176)	Others<br>(n=38)	SMD
**Angiographic and procedural characteristics**							
Limb side, Right	210 (47.1)	319 (48.7)	247 (48.7)	54 (52.9)	93 (52.8)	15 (39.5)	0.114
Procedural approach							
Ipsilateral	162 (36.3)	165 (25.2)	167 (32.9)	25 (24.5)	41 (23.3)	11 (28.9)	0.139
Contralateral	291 (65.2)	516 (78.8)	358 (70.6)	81 (79.4)	139 (79.0)	28 (73.7)	0.153
Lesion location							
Distal Aorta	44 (9.9)	118 (18.0)	58 (11.4)	15 (14.7)	30 (17.0)	5 (13.2)	0.114
Common iliac artery	44 (9.9)	120 (18.3)	58 (11.4)	16 (15.7)	31 (17.6)	6 (15.8)	0.117
External iliac artery	0 (0.0)	1 (0.2)	0 (0.0)	0 (0.0)	0 (0.0)	1 (2.6)	0.091
Common femoral artery	11 (2.5)	14 (2.1)	32 (6.3)	6 (5.9)	7 (4.0)	0 (0.0)	0.175
Superficial femoral artery	446 (100.0)	655 (100.0)	507 (100.0)	102 (100.0)	176 (100.0)	38 (100.0)	<0.001
Infra-popliteal artery	171 (38.3)	146 (22.3)	192 (37.9)	41 (40.2)	35 (19.9)	15 (39.5)	0.227
Anterior tibial artery	100 (22.4)	83 (12.7)	82 (16.2)	16 (15.7)	16 (9.1)	4 (10.5)	0.163
Posterior tibial artery	70 (15.7)	48 (7.3)	55 (10.8)	9 (8.8)	13 (7.4)	6 (15.8)	0.146

Peroneal artery	43 (9.6)	40 (6.1)	45 (8.9)	8 (7.8)	7 (4.0)	6 (15.8)	0.166
Total occlusion lesion	188 (42.2)	351 (53.6)	199 (39.3)	70 (68.6)	102 (58.0)	25 (65.8)	0.306
Calcified lesion	240 (53.8)	404 (61.7)	284 (56.0)	43 (42.2)	85 (48.3)	21 (55.3)	0.165
Moderate/Severe calcification	108 (24.2)	191 (29.2)	173 (34.1)	27 (26.5)	63 (35.8)	10 (26.3)	0.123
TASC -II (C/D)	217 (48.7)	430 (65.6)	246 (48.5)	71 (69.6)	103 (58.5)	31 (81.6)	0.339
Distal runoff vessels	2.52 (0.79)	2.74 (0.61)	2.64 (0.70)	2.68 (0.65)	2.80 (0.56)	2.58 (0.79)	0.183
Total lesion length, mm	114.83 (104.06)	135.99 (82.94)	190.70 (113.49)	317.87 (164.90)	154.17 (86.65)	113.40 (63.19)	0.720
Lesion diameter, mm (max)	5.33 (1.03)	6.48 (0.80)	5.62 (0.72)	6.50 (0.86)	6.40 (0.49)	6.80 (1.10)	0.779
Sub-intimal approach	62 (13.9)	135 (20.6)	43 (8.5)	23 (22.5)	46 (26.1)	9 (23.7)	0.214
Atherectomy	24 (5.4)	26 (4.0)	124 (24.5)	9 (8.8)	9 (5.1)	24 (63.2)	0.646
DAART	23 (5.2)	26 (4.0)	124 (24.5)	9 (8.8)	9 (5.1)	0 (0.0)	0.331

	**IPTW population**						
**Variables mean ± SD or n (%)**	**POBA (n=899)**	**BMS (n=1255)**	**DCB (n=1119)**	**DCB+BMS (n=213)**	**DES (n=351)**	**Others (n=88)**	**SMD**
**Angiographic and procedural characteristics**							
Limb side, Right	425.9 (47.4)	636.5 (50.7)	526.8 (47.1)	116.9 (54.8)	187.1 (53.3)	46.5 (52.9)	0.078
Procedural approach							
Ipsilateral	289.7 (32.2)	309.3 (24.6)	377.9 (33.8)	70.6 (33.1)	81.0 (23.1)	36.1 (41.1)	0.172

Contralateral	618.7 (68.8)	997.8 (79.5)	770.6 (68.9)	170.6 (80.0)	275.0 (78.3)	53.0 (60.3)	0.210
Lesion location							
Distal Aorta	98.7 (11.0)	230.9 (18.4)	168.1 (15.0)	28.7 (13.4)	55.0 (15.7)	12.3 (14.0)	0.085
Common iliac artery	98.7 (11.0)	232.9 (18.6)	168.1 (15.0)	29.7 (13.9)	56.0 (16.0)	13.3 (15.1)	0.083
External iliac artery	0.0 (0.0)	1.0 (0.1)	0.0 (0.0)	0.0 (0.0)	0.0 (0.0)	1.0 (1.1)	0.060
Common femoral artery	18.2 (2.0)	26.0 (2.1)	54.6 (4.9)	13.7 (6.4)	10.7 (3.0)	0.0 (0.0)	0.170
Superficial femoral artery	899.3 (100.0)	1254.9 (100.0)	1118.5 (100.0)	213.3 (100.0)	351.2 (100.0)	87.8 (100.0)	<0.001
Infra-popliteal artery	338.7 (37.7)	259.4 (20.7)	474.6 (42.4)	79.9 (37.5)	66.1 (18.8)	45.2 (51.5)	0.335
Anterior tibial artery	177.4 (19.7)	136.1 (10.8)	272.2 (24.3)	29.1 (13.6)	35.2 (10.0)	23.2 (26.4)	0.228
Posterior tibial artery	150.4 (16.7)	83.5 (6.7)	215.6 (19.3)	15.4 (7.2)	21.9 (6.2)	24.6 (28.0)	0.298
Peroneal artery	87.8 (9.8)	82.3 (6.6)	107.8 (9.6)	13.5 (6.3)	10.4 (3.0)	13.9 (15.8)	0.187
Total occlusion lesion	374.4 (41.6)	653.0 (52.0)	511.5 (45.7)	149.4 (70.0)	206.0 (58.7)	62.4 (71.0)	0.314
Calcified lesion	450.1 (50.1)	782.9 (62.4)	636.3 (56.9)	77.6 (36.4)	147.3 (41.9)	49.8 (56.7)	0.247
Moderate/Severe calcification	202.5 (22.5)	382.0 (30.4)	436.6 (39.0)	49.8 (23.3)	110.9 (31.6)	34.1 (38.8)	0.190
TASC -II (C/D)	480.6 (53.4)	820.3 (65.4)	583.3 (52.2)	160.5 (75.2)	203.2 (57.9)	77.3 (88.0)	0.382
Distal runoff vessels	2.54 (0.80)	2.76 (0.60)	2.47 (0.88)	2.73 (0.60)	2.81 (0.53)	2.30 (0.90)	0.325
Total lesion length, mm	115.46 (103.67)	134.40 (81.21)	201.41 (117.99)	325.45 (155.20)	154.29 (84.98)	112.22 (60.45)	0.789
Lesion diameter, mm (max)	5.33 (0.99)	6.51 (0.79)	5.62 (0.67)	6.45 (0.83)	6.42 (0.50)	6.65 (1.19)	0.734

Sub-intimal approach	148.6 (16.5)	242.0 (19.3)	74.9 (6.7)	50.6 (23.7)	93.3 (26.6)	18.4 (20.9)	0.224
Atherectomy	46.4 (5.2)	50.3 (4.0)	309.5 (27.7)	19.0 (8.9)	19.8 (5.6)	67.1 (76.3)	0.827
DAART	45.3 (5.0)	50.3 (4.0)	309.5 (27.7)	19.0 (8.9)	19.8 (5.6)	0.0 (0.0)	0.357

Values are presented as mean ± standard deviation or number (percentage).

SMD, standardized mean difference; DAART, directional atherectomy and antirestenotic therapy; POBA, plain old balloon angioplasty; BMS, bare-metal stent; DCB, drug-coated balloon; DES, drug-eluting stent; TASC-II=Trans-Atlantic Inter-Society Consensus II.

Supplement Table S5. Patients who experienced worsening symptom after EVT

	IC (N=477)	CLTI (N=453)	P-value
**Repeat PTA**	122 (25.6)	107 (23.6)	0.538
**Time from symptom aggravation to repeat PTA (days)**	63.0±115.4	50.2±90.4	0.368
**Amputation without EVT**	-	53 (11.7)	
**Only conservative treatment**	355 (74.4)	346 (76.4)	0.538

Values are presented as number (percentage) or mean ± standard deviation.
EVT = Endovascular treatment; PTA = Percutaneous transluminal angioplasty; CLTI = Chronic limb-threatening ischemia; IC = Intermittent claudication.
